# Early postoperative pain as a risk factor of shoulder stiffness after arthroscopic rotator cuff repair

**DOI:** 10.1186/s10195-021-00585-9

**Published:** 2021-06-26

**Authors:** Mohammad Reza Guity, Amir Sobhani Eraghi, Fatemeh Sadat Hosseini-Baharanchi

**Affiliations:** 1 Department of Orthopaedics, Imam Khomeini Hospital, Tehran University of Medical Sciences, Tehran, Iran; 2 Department of Orthopaedics, Rasool Akram Medical Complex, Iran University of Medical Sciences, Niayesh Ave., Sattarkhan St, Tehran, Iran; 3 Minimally Invasive Surgery Research Center & Department of Biostatistics, School of Public Health, Iran University of Medical Sciences, Tehran, Iran

**Keywords:** Rotator cuff tear, Arthroscopy, Risk factor, Pain

## Abstract

**Background:**

The role of postoperative pain in incidence of shoulder stiffness (SS) after shoulder arthroscopy has not been thoroughly investigated. The present study was conducted to assess the effects of early postoperative pain (EPOP) on onset of SS after arthroscopic rotator cuff (RC) repair.

**Materials and methods:**

In a retrospective analysis of a prospectively collected database, 335 patients who underwent arthroscopic RC repair were evaluated. RC tendons were sutured to the bone using the double-row technique. EPOP was evaluated 1 week after surgery using the visual analog scale (VAS). SS was assessed 3 months after surgery and was categorized into moderate or severe based on shoulder range of motion (ROM). Each type of complication including SS was identified and recorded.

**Results:**

Postoperative shoulder stiffness (POSS) was identified in 121 patients (36.2%) that was moderate in 86 patients (70.1%) and severe in 35 patients (28.9%). After 1 week, VAS pain score was equal to 7.7 ± 3.1 and 4.5 ± 2.1 in the patients with and without stiffness, respectively (*p* < 0.001). Diabetes and traumatic tear were found to be associated with postoperative stiffness (*p* = 0.046 and *p* < 0.001, respectively). Similar associations were found on multivariate analysis of data. VAS pain score was higher in the patients with severe stiffness compared with those with moderate stiffness (*p* < 0.001).

**Conclusions:**

Our findings revealed that EPOP is associated with shoulder stiffness after arthroscopic RC repair. Therefore, strategies to ameliorate EPOP could be opted to decrease rate of POSS.

**Level of evidence:**

Level IV

## Introduction

Surgical intervention is widely used for treatment of rotator cuff (RC) tear, which is generally associated with good outcomes and low rate of complications [1, 2]. Postoperative stiffness (POS) is one of the most common complications after arthroscopic RC repair [3]. Restrictions on the patients’ quality of life have led the researchers to focus their studies on risk factors of shoulder stiffness (SS). Diabetes and postoperative shoulder stiffness (POSS), as well as prolonged immobilization and poor patient compliance with postoperative rehabilitation, have been reported to be associated with POS after arthroscopic RC repair [4, 5]. Overall, risk of POS has remained at a high level of 32.7% [6].

Inability to perform early shoulder movements after arthroscopic RC repair has been suggested as a determining factor in incidence of POSS [5]. It is hypothesized that postoperative pain might cause less shoulder movement as well as the reduced compliance with rehabilitation programs, thereby increasing risk of POSS. If this hypothesis is confirmed, rate of POS could be reduced in a significant number of the patients by adequately controlling early postoperative pain (EPOP).

The role of postoperative pain in the incidence of stiffness following shoulder arthroscopy has not been thoroughly investigated. Therefore, the present study was carried out to assess the effects of postoperative pain on onset of SS after arthroscopic RC repair.

## Materials and methods

This study, which was a retrospective analysis of a prospectively collected database, was approved by the review board of our institute under the code IR.IUMS.FMD.REC.1398.471, and informed written consent was obtained from the patients to publish their medical data. During 2012–2018, patients who underwent arthroscopic RC repair were identified in our university hospital. Exclusion criteria were POSS, arthritic shoulder, revision repair, previous shoulder surgery, and concurrent spinal cord lesions. SS was categorized into moderate or severe levels according to passive range of motion (ROM) as defined in the study by Tauro et al. [7]. A deficit of 25–70° in the total passive ROM (abduction, forward elevation, external rotation, and internal rotation) was considered as moderate stiffness, while a deficit > 70° was considered as severe stiffness. Arthritic shoulders were identified according to the Walch classification system and excluded [8]. Patients with superior labral tear from anterior to posterior (SLAP) and Bankart lesion were also excluded. Patients lost to follow-up were excluded from the study as well (*n* = 13). Of 493 patients who underwent arthroscopic RC repair in our center during the study period, 335 patients met the inclusion criteria and were selected for this study.

Surgeries were performed by the same senior surgeon (ASA) on the patients in beach chair position and under general anesthesia. RC tendons were sutured to the bone using the double-row technique [9]. We performed a long head of biceps (LHB) tenotomy in patients older than 50 years of age and a tenodesis in those younger than 50 years. Tear size was assessed intraoperatively and was classified into four categories: small (<1 cm), medium (1–3 cm), large (3–5 cm), and massive (> 5 cm) [10].

The patients were discharged within 24 h after the operation. For controlling postoperative pain, pregabalin (75 mg BID) and celecoxib (200 mg BID) were administered for 2 weeks after operation. A tramadol tablet (25 mg) was administered for further pain control between days 3 and 7, in 82 (24.4%) of the patients.

The operated shoulder was immobilized in a sling for 2 weeks. Pendulum exercises and passive flexion up to 90° in sleeping position were started after 2 weeks. Active-assisted ROM exercises were started after 4 weeks. Strengthening exercises were performed after 2 months. Exercises were supervised by a musculoskeletal physiotherapist.

Follow-up visits were performed after 1 week (7.3 ± 1.6) and 3 months (95.1 ± 6.3). Pain level was evaluated using the visual analog scale (VAS), ranging from 0 (no pain) to 10 (extreme pain). Each type of complication including SS was identified and recorded.

### Statistical analysis

SPSS software for Windows version 16 (Chicago, Illinois, USA) was used for statistical analysis of the data. Descriptive data were reported as mean with standard deviation or number and percentage. Shapiro–Wilk test was used for evaluation of normality of the data. The independent-samples *t*-test or its nonparametric counterpart (Mann–Whitney *U* test) was used for comparison of mean values between two independent groups. The *χ*^2^ test was used for evaluation of statistical association between categorical variables. For eliminating the confounding effects, significant factors in univariate analysis were included in a binary regression model. A *p*-value of < 0.05 was regarded as statistically significant.

## Results

Characteristics of the patients are presented in Table 1.

**Table 1:** Characteristic features of the patients who underwent arthroscopic repair for rotator cuff tear

Variable	Patients (*n* = 335)
Age (years)	68.5 ± 9.6
Sex	
Male	128 (38.2)
Female	(61.8) 207
Laterality	
Left	117 (34.9)
Right	218 (65.1)
Etiology	
Traumatic	84 (25.1)
Atraumatic	251(74.9)
Diabetes mellitus	
Yes	46 (13.7)
No	289 (86.3)
Mean time from symptom onset to operation (month)	6.8 ± 4.9
Involved tendon	
Supraspinatus	127 (37.9)
Subscapularis	27 (8)
Supraspinatus and subscapularis	70 (20.9)
Supraspinatus and infraspinatus	74 (22.1)
Supraspinatus and subscapularis and infraspinatus	37 (11.1)
Tear size	
Small	86 (25.7)
Medium	92 (27.5)
Large	81 (24.2)
Massive	76 (22.6)
Associated procedures	
Tenotomy	311 (92.8)
Tenodesis	24 (7.2)
Postoperative stiffness	
Yes	121 (36.1)
No	214 (63.9)

Data are shown as mean ± SD or number (%)

POSS was identified in 121 patients (36.2%). After 1 week, VAS pain was equal to 7.7 ± 3.1 and 4.5 ± 2.1 in the patients with and without stiffness (*p* < 0.001), respectively. Diabetes and traumatic tear were found to be associated with incidence of POS (*p* = 0.046 and *p* < 0.001, respectively). The subgroups of the patients with and without POS were similar in terms of other characteristics, including mean age (*p* = 0.71), gender (*p* = 0.85), and tear size (*p* = 0.57) (Table 2). Predictive values of postoperative pain and other postoperative variables are reported in Table 3.

**Table 2:** Comparison of characteristic features between patients with and without postoperative stiffness

Variable	Without stiffness (*n* = 214)	With stiffness (*n* = 121)	*p*-Value
Age (years)	68.57 ± 9.9	68.28 ± 9.8	0.71
Sex			
Male	81 (38)	47 (38.8)	0.85
Female	133 (62.2)	74 (61.2)	
Laterality			
Right	140 (65.4)	78 (65.5)	0.82
Left	74 (34.6)	43 (35.5)	
Diabetes			
Yes	26 (12.1)	20 (16.5)	0.046
No	188 (87.9)	101 (83.5)	
Involved tendon			
Supraspinatus	79 (36.9)	49 (40.5)	
Subscapularis	17 (8)	8 (6.6)	
Supraspinatus and subscapularis	47 (22)	27 (22.3)	0.39
Supraspinatus and infraspinatus	51 (23.8)	27 (22.3)	
Supraspinatus and subscapularis and infraspinatus	20 (9.3)	10 (8.3)	
Tear size			
Small	52 (24.2)	34 (28.1)	
Medium	61 (28.5)	31 (25.7)	0.57
Large	53 (24.8)	28 (23.1)	
Massive	48 (22.5)	28 (23.1)	
Associated procedures			
Tenotomy	199 (92.5)	113 (93.4)	0.77
Tenodesis	16 (7.5)	8 (6.6)	
Mean time from symptom onset to operation (month)	6.7 ± 4.7	6.9 ± 5.2	0.69
Etiology			
Traumatic	26 (12)	58 (48)	< 0.001
Atraumatic	188 (88)	63 (52)	
Mean VAS 1 week after surgery	4.5 ± 2.1	7.7 ± 3.1	< 0.001

Data are shown as mean ± SD or number (%)

*VAS* visual analog scale

**Table 3:** Binary logistic regression model showing predictive value of variables for postoperative shoulder stiffness following arthroscopic repair of RCT

Variable	Odds ratio	*p*-Value	95% CI	
			Lower	Upper
VAS of 1 week after surgery	21.8	< 0.001	8.771	54.25
Diabetes mellitus	1.573	0.04	1.262	2.050
Traumatic RCT	5.32	< 0.001	2.643	10.71

*RCT* rotator cuff tear, *VAS* visual analog scale

Stiffness level was moderate in 86 patients (70.1%) and severe in 35 (28.9%). VAS pain score was higher in the patients with severe stiffness (*p* < 0.001). Rate of severe stiffness was higher in the patients with traumatic RC tear compared with those with atraumatic (*p* = 0.04). Tear size did not influence severity of stiffness (*p* = 0.41)‏ (Table 4).

**Table 4:** Comparison of characteristic features between patients with moderate and severe postoperative stiffness

Variable	Moderate stiffness (*n* = 86)	Severe stiffness (*n* = 35)	*p*-Value
Age (years)	67.9 ± 8.8	69.2 ± 9.9	0.31
Sex			
Male	33 (38.4)	14 (40)	0.51
Female	53 (61.6)	21 (60)	
Laterality			
Right	57 (66.3)	23 (65.7)	0.66
Left	29 (33.7)	12 (34.3)	
Diabetes			
Yes	19 (22)	7 (20)	0.42
No	67 (78)	28 (80)	
Involved tendon			
Supraspinatus	36 (41.9)	13 (37.1)	
Subscapularis	5 (5.8)	3 (8.6)	
Supraspinatus and subscapularis	19 (22.1)	8 (22.9)	0.54
Supraspinatus and infraspinatus	18 (20.9)	9 (25.7)	
Supraspinatus and subscapularis and infraspinatus	8 (9.3)	2 (5.7)	
Tear size			
Small	25 (29.1)	9 (25.7)	
Medium	21 (22.1)	10 (28.6)	0. 41
Large	19 (24.4)	9 (25.7)	
Massive	21 (24.4)	7 (20)	
Associated procedures			
Tenotomy	80 (93)	33 (94.3)	0.75
Tenodesis	6 (7)	2 (5.7)	
Mean time from symptom onset to operation (months)	6.6 ± 4.5	6.8 ± 4.9	0.46
Etiology			
Traumatic	39 (45.3)	19 (54.3)	0.04
Atraumatic	47 (54.7)	16 (46.7)	
Mean VAS 1 week after surgery	7.4 ± 2.9	8.3 ± 3.1	< 0.001
Mean VAS 3 months after surgery	7.7 ± 2.8	8.8 ± 3.5	< 0.001

Data are shown as mean ± SD or number (%)

*VAS* visual analog scale

## Discussion

Results of this study showed that postoperative pain was a risk factor for development of SS after arthroscopic RC repair and was strongly associated with severity of stiffness. Diabetes and traumatic onset of RC tear were found as additional risk factors of POSS. These findings have certain implications in the clinical practice and need to be considered.

Namdari et al. revealed that early postoperative limitation in ROM is associated with a higher risk of POSS [11]. The role of early postoperative motion to prevent SS has been discussed in other investigations as well [5]. Low compliance with postoperative rehabilitation programs has also been frequently reported as a risk factor of POSS [12–14]. On the other hand, worsening pain during exercise presents a barrier to following exercises. Accordingly, it can be said that SS in the patients with severe postoperative pain is caused by the decreased compliance with rehabilitation program. Thus, controlling of postoperative pain might reduce rate of SS after arthroscopic repair of RC.

Nonsteroidal antiinflammatory drugs (NSAIDs) were used for controlling postoperative pain in the present cohort. These medications potentially contribute to reduction of the pain and stiffness through their antiinflammatory activity [15]. However, postoperative stiffness was still observed in a significant number of our patients. Recent studies have suggested corticosteroid injection as a safe and efficacious modality for treatment of persistent postoperative shoulder pain during the recovery phase [16, 17].

Herein, all the surgeries were performed under general anesthesia. However, a combination of general and regional anesthesia (interscalene block) is known to facilitate postoperative pain management in shoulder surgeries [18]. Therefore, supplemental regional anesthesia could be used to reduce postoperative pain and its potential subsequent stiffness.

Similar to our study, Namdari et al. introduced diabetes mellitus as a risk factor of SS [11]. Diabetic stiff hand syndrome, also known as diabetic cheiroarthropathy, is a condition characterized by alterations in collagen production, breakdown, and composition, leading to limited mobility of joints in the hands and fingers, presented as flexion contractures [19]. Therefore, the role of diabetes as a risk factor of POSS should also be taken into account.

Huberty et al. reported SS in 24 (4.9%) out of 489 patients who underwent arthroscopic RC repair. According to their results, patients less than 50 years of age and with larger tears were less likely to develop SS after arthroscopic RC repair [20]. Chung et al. reported POSS in 27.8% of patients (80/288). In this study, contrary to the study by Huberty et al., older age and larger size of the tear were found to be associated with a higher risk of POS [21]. Age and tear size were not significantly associated with incidence or severity of stiffness in the present series.

Seo et al. evaluated risk factors of POSS in 119 patients who underwent arthroscopic RC repair. Based on their results, a higher percentage of stiffness was seen in full-thickness tears in comparison with a partial-thickness tear. In addition, patients with a traumatic tear had a statistically higher rate of POSS [22]. Herein, the patients with partial-thickness tears were not included. However, the role of partial thickness in genesis of pain is controversial, in that, sometimes, small and partial tears are more likely to lead to POS than large tears [23]. Our findings showed that traumatic RC tear was a risk factor of POSS, in line with the study by Seo et al. [22]. This is probably due to the presence of associated injuries in traumatic RC tear, such as acromioclavicular joint injury, labral injuries, bruise or fracture of shoulder bones, strain of intrinsic and extrinsic shoulder muscles, and neurologic injuries to the axillary and/or suprascapular nerve.

Several studies have investigated the risk factors of SS after arthroscopic RC repair [24–26]; however, studies exploring the role of postoperative pain in the onset of SS are lacking. Basic science research has investigated the role of different inflammation mediators in genesis of pain in the case of SS‏. In this respect, overexpression of inflammatory cytokines, such as interleukin 1 (IL-‏‏1), interleukin 6 (IL-6), tumor necrosis factor-alpha (TNF-α), and substance P, may indirectly increase risk of POSS ‏through increasing local pain level and causing limited ROM. Mediators such as substance P ‏might also directly induce incidence of POSS through initiating a fibrotic cascade by stimulation of transforming growth factor-beta (TGF-β) expression [5, 27].

Management of POSS after arthroscopic RC repair is a controversial issue [24]. The need for joint immobilization to preserve biologic healing of the RC may be implicated in the development of POS [24]. Yet‏, several other factors might contribute to this pathogenesis.‏ The type of technique used for RC repair can influence the level of POS, and arthroscopic repair has been reported to be associated with less POS than open repair [28, 29]. Single-tendon tears have been reported more likely to develop POSS than multiple-tendon involvement [20]. Moreover, postoperative pain and stiffness has been associated with repair of subscapularis tear, accounting for up to 53% of the cuff movements [30, 31]. Associated procedures LHB tenotomy or tenodesis, acromioplasty, and capsulotomy [32], and glenohumeral/acromioclavicular osteoarthritis could also increase the rate of POSS [29, 33, 34].

This study has some limitations. The retrospective design and the data of only two follow-up time-points are the main limitations of the study.

## Conclusion

EPOP seems to play a prominent role in incidence of SS after arthroscopic RC repair, probably through reducing compliance with rehabilitation programs. Appropriate strategies of postoperative pain control could significantly reduce the risk to develop a stiff shoulder.

## Data Availability

The datasets generated and/or analyzed during the current study are available from the corresponding author on reasonable request.

## Abbreviations

RCT: Rotator cuff tear
ROM: Range of motion
VAS: Visual analog scale
EPOP: Early postoperative pain
SS: Shoulder stiffness
RC: Rotator cuff
POSS: Postoperative shoulder stiffness
POS: Postoperative stiffness
SLAP: Superior labral tear from anterior to posterior
LHB: Long head of biceps
NSAIDs: Nonsteroidal antiinflammatory drugs
